# Calcineurin/NFAT Activation-Dependence of Leptin Synthesis and Vascular Growth in Response to Mechanical Stretch

**DOI:** 10.3389/fphys.2016.00433

**Published:** 2016-09-29

**Authors:** Nadia Soudani, Crystal M. Ghantous, Zein Farhat, Wassim N. Shebaby, Kazem Zibara, Asad Zeidan

**Affiliations:** ^1^Department of Anatomy, Cell Biology and Physiology, American University of BeirutBeirut, Lebanon; ^2^Department of Natural Sciences, Lebanese American UniversityByblos, Lebanon; ^3^Laboratory of Stem Cells, Department of Biology, Faculty of Sciences, Lebanese UniversityBeirut, Lebanon

**Keywords:** leptin, hypertension, hypertrophy, calcineurin, NFAT

## Abstract

**Background and Aims:** Hypertension and obesity are important risk factors of cardiovascular disease. They are both associated with high leptin levels and have been shown to promote vascular hypertrophy, through the RhoA/ROCK and ERK1/2 phosphorylation. Calcineurin/NFAT activation also induces vascular hypertrophy by upregulating various genes. This study aimed to decipher whether a crosstalk exists between the RhoA/ROCK pathway, Ca^2+^/calcineurin/NFAT pathway, and ERK1/2 phosphorylation in the process of mechanical stretch-induced vascular smooth muscle cell (VSMC) hypertrophy and leptin synthesis.

**Methods and Results:** Rat portal vein (RPV) organ culture was used to investigate the effect of mechanical stretch and exogenous leptin (3.1 nM) on VSMC hypertrophy and leptin synthesis. Results showed that stretching the RPV significantly upregulated leptin secretion, mRNA, and protein expression, which were inhibited by the calcium channel blocker nifedipine (10 μM), the selective calcineurin inhibitor FK506 (1 nM), and the ERK1/2 inhibitor PD98059 (1 μM). The transcription inhibitor actinomycin D (0.1 μM) and the translation inhibitor cycloheximide (1 mM) significantly decreased stretch-induced leptin protein expression. Mechanical stretch or leptin caused an increase in wet weight changes and protein synthesis, considered as hypertrophic markers, while they were inhibited by FK506 (0.1 nM; 1 nM). In addition, stretch or exogenous leptin significantly increased calcineurin activity and MCIP1 expression whereas leptin induced NFAT nuclear translocation in VSMCs. Moreover, in response to stretch or exogenous leptin, the Rho inhibitor C3 exoenzyme (30 ng/mL), the ROCK inhibitor Y-27632 (10 μM), and the actin depolymerization agents Latrunculin B (50 nM) and cytochalasin D (1 μM) reduced calcineurin activation and NFAT nuclear translocation. ERK1/2 phosphorylation was inhibited by FK506 and C3.

**Conclusions:** Mechanical stretch-induced VSMC hypertrophy and leptin synthesis and secretion are mediated by Ca^2+^/calcineurin/NFAT activation. RhoA/ROCK and ERK1/2 activation are critical for mechanical stretch-induced calcineurin activation.

## Introduction

Hypertension affects blood vessels of various organs, making them stiff, less elastic, and at risk of blockage or rupture (Lifton et al., 2001). It is a major risk factor for many cardiovascular diseases such as aneurysms, atherosclerosis, stroke, left ventricular hypertrophy, and vascular hypertrophy (Intengan and Schiffrin, 2001; Kuhlencordt et al., 2001; Hoenig et al., 2008). The force imposed by high blood pressure mechanically stretches blood vessels circumferentially, which in turn promotes vascular smooth muscle cell (VSMC) remodeling in the form of hypertrophy (Zeidan et al., 2005; Shyu, 2009). A reliable model for mimicking hypertension *in vitro* is to mechanically stretch the rat portal vein (RPV), which has spontaneous contractile activity and longitudinally-oriented VSMCs (Sutter, 1990). This low-pressure blood vessel is sensitive to pressure increase and undergoes hypertrophy when under hypertensive conditions (Malmqvist and Arner, 1988, 1990; Zeidan et al., 2000). It has also been used as an analog for small pre-capillary resistance blood vessels (Ljung, 1990; Sutter, 1990). Thus, mechanically stretching the RPV with a specific force that mimics the force of stretch (See Rat Portal Vein Organ Culture) during hypertension is a well-characterized system that mimics hypertension in order to study the hypertrophic effect of stretch (Zeidan et al., 2000, 2003a,b, 2005; Ren et al., 2010; Turczynska et al., 2012).

We have previously shown that in mechanical stretch-induced VSMC hypertrophy, G-actin levels are lowered compared to F-actin levels due to changes in the actin cytoskeleton dynamics via the RhoA/ROCK pathway (Zeidan et al., 2003b, 2006, 2007). The PI3K/AKT pathway also induces changes in the actin cytoskeleton through phosphorylation of LIMK/cofilin (Zeidan et al., 2007). Moreover, MAP kinases, such as ERK1/2 and p38, play a significant role in promoting VSMC hypertrophy as a result of mechanical stretch (Zeidan et al., 2000, 2003a). We have also demonstrated that mechanical stretch induces the secretion of the obesity-associated hormone leptin from VSMCs (Maffei et al., 1995) and upregulates leptin mRNA expression after 1–3 days of stretch (Zeidan et al., 2005).

Leptin is a 16 kDa protein that is the product of the *ob* gene (Zeidan and Karmazyn, 2006) and found in excessive levels in obesity (Sinha et al., 1996). It reduces appetite and increases energy expenditure, but also found to exert pleiotropic effects on several physiological systems, such as the nervous, immune, reproductive, and cardiovascular systems (Zeidan et al., 2006; Karmazyn et al., 2008; Fernández-Riejos et al., 2010; Zuure et al., 2013; Procaccini et al., 2014; reviewed by Ghantous et al., 2015a). Leptin also plays a detrimental role in the development of several obesity-associated cardiovascular diseases (Margetic et al., 2002; Rahmouni and Haynes, 2004) such as atherosclerosis (Schëfer et al., 2004; Schneiderman et al., 2012), left ventricular hypertrophy (Perego et al., 2005; Zeidan et al., 2006), and vascular hypertrophy (Zeidan et al., 2005). Several studies have shown that hypertension is associated with high plasma levels of leptin (Agata et al., 1997; Hiraoka et al., 1997; Stenvinkel, 2000). We have shown that blood vessels under mechanical stretch, a model mimicking hypertension, have the ability to produce and secrete leptin protein (Zeidan et al., 2005; Ghantous et al., 2015b) and leptin receptor mRNA expression (Zeidan et al., 2005). Leptin has also been shown to activate the Ca^2+^/calmodulin-dependent phosphatase calcineurin, which in turn promotes cardiomyocyte hypertrophy (Rajapurohitam et al., 2012). However, the exact mechanisms by which mechanical stretch induces VSMC leptin synthesis and hypertrophy and whether calcineurin is involved in this process have not been fully elucidated yet. We hypothesized that calcineurin and nuclear factor of activated T cells (NFAT) are key intermediates in this pathway.

NFAT is a family of transcription factors (NFAT 1–5; Rao et al., 1997; Feske et al., 2003; Pang and Sun, 2009) found in the cytosol in the phosphorylated, inactive form (Kudryavtseva et al., 2013). Calcineurin, Ca^2+^/calmodulin-dependent serine/threonine phosphatase, dephosphorylates NFAT leading to its activation (Rao et al., 1997; Kudryavtseva et al., 2013) and translocation to the nucleus, which activates gene transcription (Hill-Eubanks et al., 2003). In skeletal muscle cells, NFAT upregulates expression of genes involved in differentiation, maturation (Delling et al., 2000), and hypertrophy (Musarò et al., 1999; Horsley et al., 2001), whereas it promotes cardiac hypertrophy in the heart (Molkentin et al., 1998; Bueno et al., 2002a; Fiedler et al., 2002; Hullmann et al., 2014). In addition, NFAT mediates VSMC proliferation (Pang and Sun, 2009; Kudryavtseva et al., 2013), hypertrophy (Suzuki et al., 2002), and angiogenesis (Graef et al., 2001; Gomez et al., 2002). However, the exact mechanisms by which calcineurin/NFAT promote hypertrophy of VSMCs have not been fully uncovered yet. Therefore, we hypothesized that leptin and the RhoA/ROCK pathway belong to the pathway of calcineurin/NFAT-induced VSMC hypertrophy.

To date, neither calcineurin signaling nor NFAT activation and nuclear translocation have been linked to mechanical stretch/leptin-mediated vascular remodeling. Accordingly, the present study was designed to take a closer look at the mechanisms of mechanical stretch/leptin-induced vascular remodeling and identify the role and the interaction of the RhoA/ROCK pathway and calcineurin/NFAT activation in this process.

## Materials and methods

### Rat portal vein organ culture

Two hundred to two hundred fifty grams of Sprague-Dawley male rats were euthanized by CO_2_, as approved by the Animal Ethics Committee, American University of Beirut. In a sterile environment, RPVs were dissected, divided longitudinally into two strips, and weighed when the experiments required. They were cultured either unloaded or loaded with 0.6 g weights (stretch the RPV slightly above optimal length or 10% stretch) in DMEM F-12 HAM culture media with 5% penicillin/streptomycin. The loads were mounted on the veins using 6-0 suture silk strings to hold the weights. They were then incubated at 37°C, 5% CO_2_ in air. When leptin (Rat Leptin, Biovision, San Francisco, USA) was used in the experiment, a concentration of 3.1 nM was added, equivalent to the average concentration found in obesity (Maffei et al., 1995). In experiments that measured changes in wet weight, RPVs were gently blotted using filter paper and then weighed after organ culture, and the difference between the wet weights was calculated as previously described (Zeidan et al., 2000).

Inhibitors such as the transcription inhibitor actinomycin D (0.1 μM; Sigma Aldrich, Missouri, USA), the translation inhibitor cycloheximide (1 mM; Sigma Aldrich, Missouri, USA), the actin depolymerization agents cytochalasin D (1 μM, Calbiochem, California, USA), latrunculin B (50 nM, Sigma Aldrich, Missouri, USA), the selective Rho inhibitor clostridial toxin C3 exoenzyme (30 ng/mL, Alexis Biochemicals, Carlsbad, California, USA), the selective ROCK inhibitor Y-27632 (10 μM, Sigma Aldrich, Missouri, USA), the selective calcineurin inhibitor FK506 (0.1 or 1 nM, Sigma Aldrich, Missouri, USA), the selective ERK inhibitor PD98059 (1 μM, Sigma Aldrich, Missouri, USA), and the calcium channel blocker nifedipine (1 or 10 μM, Sigma Aldrich, Missouri, USA) were added to the media 60 min before mechanically stretching the RPV or adding leptin. Following incubation, the RPV strips were taken out of the incubator and either weighed (Zeidan et al., 2000) or immediately frozen in liquid nitrogen and stored at −80°C for protein analysis.

### Immunoblotting

RPVs were homogenized using lysis buffer (50 mM Tris, pH = 8.0, 150 mM NaCl, 1% Nonidet-P40, 0.5% sodium deoxycholate) and liquid nitrogen. Proteins were extracted following centrifugation of the samples for 10 min at 4°C and quantified using Bradford assay. Leptin protein expression and ERK1/2 phosphorylation were determined by Western blot as described previously (Zeidan et al., 2006). Primary antibodies for leptin [anti-leptin antibody Ob (Y-20)], GAPDH, P-ERK1/2, and T-ERK1/2 were added to the nitrocellulose membranes at 1:1000 ratio (3% BSA) for 1 h. All antibodies were purchased from Santa Cruz Biotechnology, California, USA.

### Protein synthesis measurement

RPVs were either stretched or incubated with leptin and then treated with FK506 (0.1 or 1 nM) for 2 days. They were then cultured for another day with [^3^H] leucine to measure protein synthesis. The radioactivity of [^3^H]-leucine incorporation into proteins was determined by liquid scintillation counting as previously described (Zeidan et al., 2003b).

### RNA isolation and real-time PCR

RNA isolation and Real-Time PCR were done as described previously (Zeidan et al., 2005). The primers were: MCIP1 forward 5′-GCCCAATCCAGACAAACAGT-3′ and MCIP1 reverse 5′-TGATTTTTGGCTTGGGTCTC-3′, leptin forward 5′-GAGACCTCCTCCATGTGCTG-3′ and leptin reverse 5′-CATTCAGGGCTAAGGTCCAA-3′, 18S rRNA forward 5′-GTAACCCGTTGAACCCCATT-3′ and 18S rRNA reverse 5′-CCATCCAATCGGTAGTAGCG-3′ which functioned as the housekeeping gene to normalize expression.

### Immunohistochemistry

To view leptin expression in RPVs, frozen sections (thickness = 5 μm) were incubated in 4% formaldehyde and permeabilized with 0.2% Triton X-100. To block non-specific binding, 1% BSA, 0.1% Triton X-100 in PBS was added for 10 min. Anti-leptin antibody (Ob Y-20, Santa Cruz Biotechnology, California, USA) was then added at 1:100 ratio in 1% BSA, PBS, and 0.05% Tween for 1 h. Finally, RPV sections were incubated with Alexa 594-conjugated goat anti-rabbit secondary antibody (1:250; Molecular Probes) for 1 h. Leptin was visualized and images were taken using a laser confocal microscope (LSM710, ZEN confocal software Carl Zeiss).

### Determination of leptin release

To determine the effect of mechanical stretch on leptin release from VSMCs, conditioned media were analyzed for leptin by a TiterZyme enzyme immunometric assay kit (Assay Designs, Inc., Ann Arbor, Michigan, USA).

### Calcineurin phosphatase activity assay

Calcineurin activity was measured by commercially available kits according to the instructions of manufacturer (Enzo Life Sciences, Plymouth Meeting, Pennsylvania).

### Determination of NFAT nuclear translocation

To study the nuclear translocation of NFAT, Rat Aortic Smooth Muscle Cells (RASMCs) were cultured (40 × 10^3^ per ml) in DMEM media with 10% fetal bovine serum for 3 days and then starved for another day. RASMCs were then treated with leptin (3.1 nM) with or without inhibitors. They were then fixed with freshly prepared 4% paraformaldehyde for 10 min, washed twice with PBS, permeabilized with 0.2% Triton X-100 in PBS with for 20 min, blocked with 1% BSA, 0.1% Triton X-100 in PBS for 10 min, and washed with PBS as described previously (Zeidan et al., 2006). They were then incubated with NFATc3 antibody (1:100 ratio, Santa Cruz Biotechnology, California, USA) overnight at 4°C followed by secondary antibody Alexa Fluor 594 goat anti-rabbit IgG (1:250 ratio, Santa Cruz Biotechnology, California, USA) for 1 h in darkness at room temperature. To stain F-actin, Phalloidin-Fluorescin isothiocyanate [phalloidin-(FITC); 1 μg/mL, Acti-stain 555 phalloidin, Cytoskeleton, Denver, CO, USA] was added for 20 min. VSMCs were then mounted on glass slides using Mounting Medium (Santa Cruz Biotechnology, California, USA) which contains the nuclear stain DAPI. NFATc3 nuclear translocation was assessed using a laser confocal microscope (LSM710, ZEN confocal software Carl Zeiss).

### Statistical analysis

Experimental group values were normalized to the unstretched and untreated RPVs. Data values are presented as mean ± standard error of the mean (S.E.M). Statistical analysis was performed using *t*-test or one-way analysis of variance (ANOVA). Statistical significance was established by Holm-Sidak or Tukey tests and considered significant for *p* < 0.05 between groups.

## Results

### Mechanical stretch induces leptin mRNA and protein expression in VSMCs

**Figure 1:**
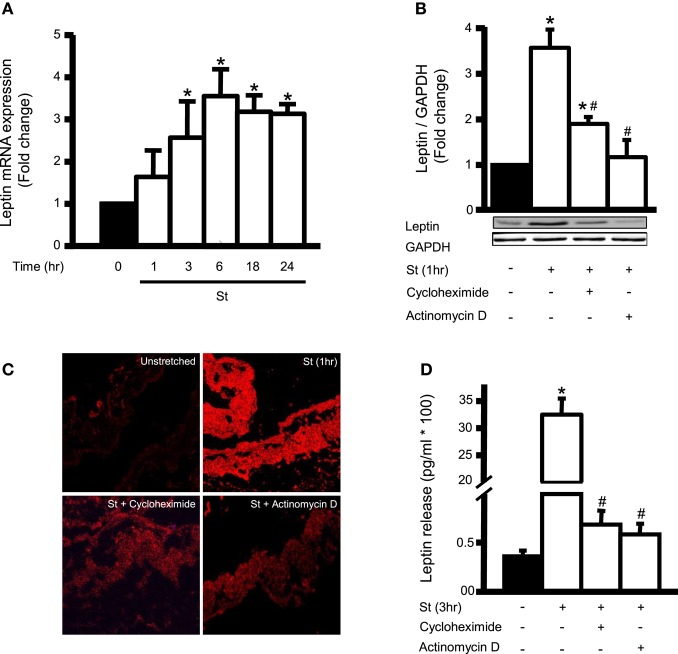
**Mechanical stretch upregulates endogenous leptin protein synthesis and is dependent on leptin RNA and protein synthesis. (A)** Effect of mechanical stretch for 1, 3, 6, 18, or 24 h on the mRNA expression of leptin (*n* = 9–11). ^*^p < 0.05 vs. unstretched (0 min). **(B)** RPVs were pre-treated with the inhibitor of transcription actinomycin D (0.1 μM) or the inhibitor of translation cycloheximide (1 mM), followed by mechanical stretch for 1 h (St 1 h). Proteins were extracted, quantified, separated by SDS-PAGE, and immunoblotted using anti-leptin and anti-GAPDH antibodies. Leptin expression was normalized using GAPDH and fold change was normalized to the unstretched RPVs. **(C)** Representative microscopic images (*n* = 4) for leptin detection in RPV wall after being mechanically stretched for 1 h and pre-treated with actinomycin D (0.1 μM) or cycloheximide (1 mM). Leptin primary antibody was detected by Alexa 594-conjugated secondary antibody (red). **(D)** Leptin release into the extracellular media was measured using ELISA for mechanically stretched RPVs for 3 h pre-treated with actinomycin D (0.1 μM) or cycloheximide (1 mM). Values are represented as mean ± S.E.M and normalized to unstretched RPVs (*n* = 7–9). ^*^p < 0.05 vs. unstretched (0 min). ^#^*p* < 0.05 vs. St.

We investigated the effect of mechanical stretch for 1, 3, 6, 18, and 24 h on leptin mRNA expression. Figure 1A shows that leptin mRNA expression increased starting 1 h (60% over basal) and remained elevated at 24 h. To ascertain whether mechanical stretch-induced leptin protein expression was mediated by mRNA or protein synthesis, RPVs were pre-treated with either actinomycin D (0.1 μM) or cycloheximide (1 mM) and mechanically stretched for 1 h (peak of leptin protein expression), followed by Western blot analysis or immunohistochemistry. Actinomycin D and cycloheximide each significantly reduced mechanical stretch-induced leptin protein expression, in comparison to untreated stretched RPVs, as shown by both Western blot and immunohistochemistry (Figures 1B,C). Thus, blocking either transcription or translation significantly reduced leptin expression in VSMCs in response to mechanical stretch, indicating that the latter activates leptin protein synthesis at the level of both transcription and translation after only 1 h of stretch.

Since we have shown that stretch induces leptin release into the medium at a peak after 3 h (Ghantous et al., 2015b), we studied the effect of actinomycin D (0.1 μM) and cycloheximide (1 mM) on mechanical stretch-induced leptin secretion after 3 h of stretch. Both compounds significantly inhibited leptin release into the culture medium (Figure 1D), indicating that the secreted leptin was synthesized by the VSMCs in response to stretch.

### Role of extracellular Ca^2+^ inflow in mechanical stretch-induced VSMC hypertrophy and leptin synthesis

**Figure 2:**
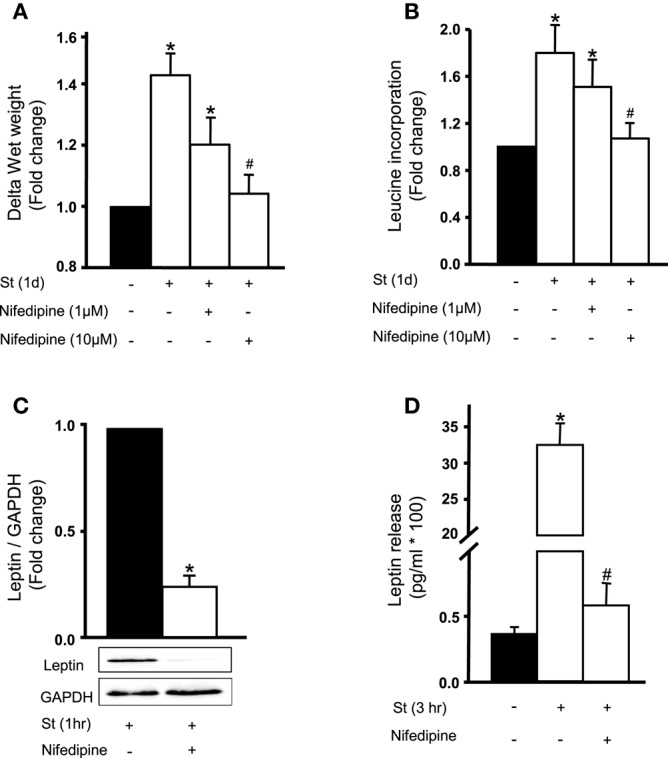
**Role of extracellular calcium in the mechanical stretch-mediated VSMC hypertrophy and leptin release**. Nifedipine (1 or 10 μM) inhibits mechanical stretch-induced **(A)** wet weight change and **(B)** protein synthesis in RPV. Nifedipine (10 μM) significantly attenuated **(C)** leptin synthesis and **(D)** release after mechanical stretch for 1 h in RPV (*n* = 6–8). ^*^p < 0.05 vs. unstretched. ^#^*p* < 0.05 vs. St.

To investigate the role of extracellular Ca^2+^ on mechanical stretch-induced RPV hypertrophy (wet weight and protein synthesis) and leptin synthesis and secretion, RPVs were incubated in Ca^2+^-depleted medium with the addition of 1 mM EGTA. Results showed that the hypertrophic effect of mechanical stretch was abolished in calcium-depleted medium (data not shown). Inhibition of Ca^2+^ inflow was then examined in mechanical stretch-induced hypertrophy using nifedipine (1 or 10 μM). Results showed that 10 μM nifedipine significantly inhibited mechanical stretch-induced increase in wet weight and protein synthesis (Figures 2A,B). These findings confirm the involvement of extracellular Ca^2+^ inflow in the mechanical stretch mediated VSMC hypertrophy.

To assess the role of calcium flow on mechanical stretch-induced leptin synthesis, RPVs were incubated for 1 h with 10 μM nifedipine followed by 1 h of stretch. Figure 2C shows that nifedipine significantly attenuated mechanical stretch-induced leptin expression in VSMCs. To study the effect of nifedipine on the release of leptin in response to stretch, RPVs were pre-treated with nifedipine and stretched for 3 h, the peak of leptin release (Ghantous et al., 2015b). Nifedipine significantly reduced mechanical stretch-induced leptin release into medium (Figure 2D). These results suggest that Ca^2+^ inflow is required for mechanical stretch-induced leptin synthesis and release from VSMCs.

### Mechanical stretch/leptin induces calcineurin activity and MCIP1 expression in VSMCs

We have previously established that mechanical stretch and leptin promote VSMC hypertrophy (Zeidan et al., 2005), similar to calcineurin (Suzuki et al., 2002). However, whether there is a crosstalk between calcineurin and mechanical stretch/leptin-induced VSMC hypertrophy has not been established yet.

**Figure 3:**
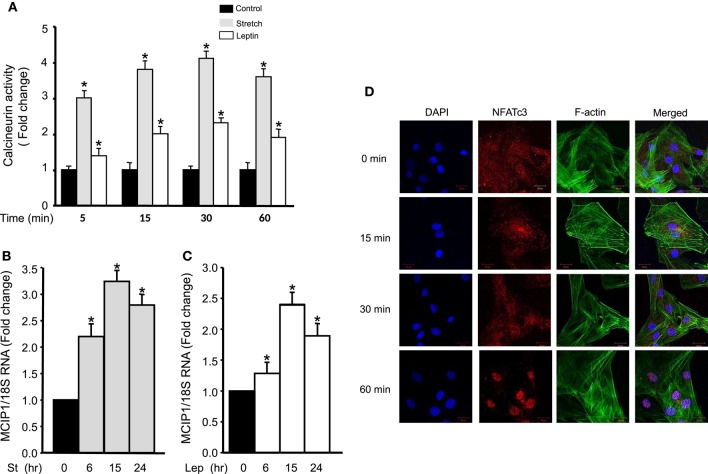
**Mechanical stretch/leptin activate calcineurin/NFAT and MCIP1 gene expression. (A)** RPVs were either mechanically stretched or treated with leptin (3.1 nM) for 5, 15, 30, or 60 min, followed by measuring the phosphatase activity of calcineurin, using a kit. Mechanical stretch and leptin each significantly activated calcineurin, with peak activation after 30 min (*n* = 8–9). ^*^p < 0.05 vs. control. **(B)** RPVs were stretched for 6, 15, or 24 h followed by Real-Time PCR. MCIP1 mRNA expression was significantly increased in response to mechanical stretch. (9–10). ^*^p < 0.05 vs. unstretched (0 h). **(C)** RPVs treated with leptin (3.1 nM) for 6, 15, or 24 h significantly upregulated MCIP1 mRNA expression. (8–10). ^*^p < 0.05 vs. unstretched (0 h). **(D)** Representative laser confocal microscopic images of RASMCs to detect NFATc3 nuclear translocation. DAPI stained the nuclei (left panel; blue). NFATc3 primary antibody was detected by Alexa Fluor 594 goat anti-rabbit secondary antibody (second panel; red). Phalloidin-FITC stained F-actin green (third panel). The overlay of all three signals is shown in the right panel (*n* = 4). Time course of leptin treatment showed that 1 h of leptin treatment activated NFAT and promoted its nuclear translocation.

To study the effect of mechanical stretch on calcineurin activation, calcineurin activity was investigated by detecting its phosphatase activity using a commercially available kit after RPVs were stretched for 5, 15, 30, and 60 min. Calcineurin activity was increased significantly after mechanical stretch for 5, 15, 30, or 60 min, with peak activation at 30 min, compared to unstretched RPVs (Figure 3A). To examine the effect of exogenous leptin on calcineurin activation, RPVs were treated with leptin (3.1 nM) for 5, 15, 30, and 60 min followed by analysis of calcineurin phosphatase activity. As shown in Figure 3A, calcineurin activity was increased significantly by exogenous leptin after 5, 15, 30, or 60 min of treatment, with peak calcineurin activation after 30 min (Figure 3A). Calcineurin activitation was higher in response to mechanical stretch than to leptin. This may be due to other mediators involved in the response to stretch, besides leptin, that are activating calcineurin.

The modulatory calcineurin-interacting protein 1 (MCIP1) gene is upregulated by calcineurin signaling and activation (Yang et al., 2000; Bush et al., 2004). To study whether mechanical stretch upregulates MCIP1 gene expression, Real-Time PCR analysis was performed on RPVs mechanically stretched for 6, 15, or 24 h. The mRNA expression of MCIP1 significantly increased after 6, 15, and 24 h of stretch, with peak expression after 15 h (Figure 3B), further indicating mechanical stretch-induced calcineurin activation.

To investigate whether leptin had a similar effect on MCIP1 gene expression, RPVs were treated with leptin for 6, 15, or 24 h, followed by Real-Time PCR analysis. Figure 3C reveals that leptin also significantly increased MCIP1 mRNA expression after 6, 15, and 24 h of leptin treatment, with peak expression after 15 h.

### Leptin induces NFAT nuclear translocation

Since NFAT is activated by calcineurin (Rao et al., 1997; Kudryavtseva et al., 2013) and is translocated to the nucleus to activate gene expression (Hill-Eubanks et al., 2003), the effect of leptin on NFATc3 activation and thus nuclear translocation in RASMCs was then investigated. Cells were treated with leptin for 15, 30, or 60 min. Results showed that NFATc3 was scattered inside the cytoplasm in untreated RASMCs. On the other hand, leptin treatment for 15 or 30 min caused a slight NFATc3 translocation to the nucleus while leptin treatment for 60 min induced full NFATc3 translocation (Figure 3D). These data indicate that leptin-induced activation of calcineurin promotes the activation of NFATc3.

### Involvement of the RhoA/ROCK pathway in leptin/mechanical stretch-induced calcineurin/NFAT activation

We have previously shown the involvement of RhoA/ROCK pathway in mechanical stretch-induced VSMC hypertrophy (Zeidan et al., 2003b). Since calcineurin/NFAT activation is implicated in VSMC hypertrophy (Suzuki et al., 2002) and since mechanical stretch activates calcnineurin/NFAT, the RhoA/ROCK pathway was then investigated for its involvement in mechanical stretch-induced calcineurin/NFAT activation. RPVs were pre-treated with either the selective RhoA inhibitor C3 (30 ng/mL) or the selective ROCK inhibitor Y-27632 (10 μM) followed by mechanical stretch for 15 min. Calcineurin activity was then measured by detecting its phosphatase activity.

**Figure 4:**
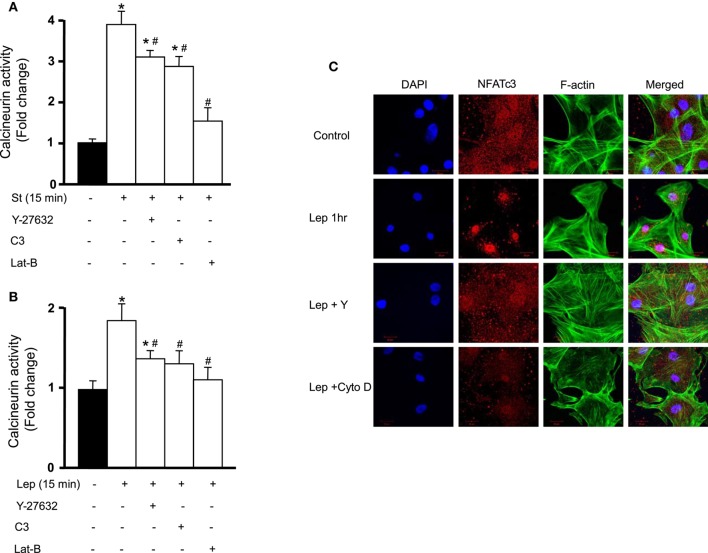
**Calcineurin/NFAT activation is mediated by the RhoA/ROCK pathway. (A)** RPVs were pre-treated with either the selective RhoA inhibitor C3 (30 ng/mL) or the selective ROCK inhibitor Y-27632 (10 μM) and mechanically stretched for 15 min. Calcineurin activity was measured by detecting its phosphatase activity. C3 and Y-27632 compound each significantly reduced calcineurin activity. The actin depolymerization agent latrunculin B (Lat-B; 50 nM) also significantly decreased stretch-induced activation of calcineurin (*n* = 8). ^*^p < 0.05 vs. unstretched. #*p* < 0.05 vs. St 15 min. **(B)** C3, Y-27632, and Lat-B each significantly reduced calcineurin activation in response to leptin (3.1 nM) treatment for 15 min (*n* = 8). ^*^p < 0.05 vs. untreated. ^#^*p* < 0.05 vs. Lep 15 min. **(C)** Laser confocal microscopic images (*n* = 4) of RASMCs reveal that NFAT nuclear translocation in response to 1 h leptin treatment (3.1 nM) was abolished by Y-27632 compound and cytochalasin D (Cyto D; 1 μM).

Treatment with either C3 or Y-27632 significantly decreased calcineurin activity, although it remained high (Figure 4A). When actin depolymerization agent latrunculin B (50 nM) was used followed by stretch for 15 min, the activity of calcineurin decreased very significantly, reaching almost negative control (unstretched) levels (Figure 4A). These results indicate that mechanical stretch-induced activation of calcineurin is mediated by the RhoA/ROCK pathway and requires an intact actin cytoskeleton.

Since leptin activates calcineurin/NFAT (Figures 3A,D), the involvement of the RhoA/ROCK pathway in calcineurin/NFAT activation in response to leptin was then studied. Pre-treatment with either C3 or Y-27632 followed by leptin treatment for 15 min resulted in a significant decrease in calcineurin activity (Figure 4B). Latrunculin B followed by 15 min of leptin treatment also significantly reduced calcineurin activity, to almost control levels (Figure 4B).

To examine the involvement of the RhoA/ROCK pathway on NFAT activation and translocation after leptin treatment for 1 h, Y-27632 (10 μM) and the actin depolymerization agent cytochalasin D (1 μM) were used followed by immunocytochemistry to visualize NFAT nuclear translocation in RASMCs. Y-27632 abolished leptin-induced NFAT translocation, as did cytochalasin D (Figure 4C). These data indicate that the RhoA/ROCK pathway and an intact actin cytoskeleton are required in leptin-induced calcineurin/NFAT activation (Figures 4B,C).

### Calcineurin promotes mechanical stretch/leptin-induced VSMC hypertrophy

**Figure 5:**
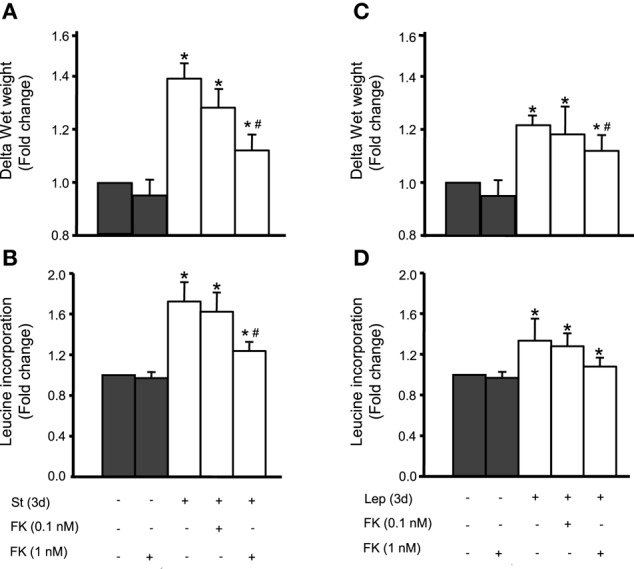
**Calcineurin is involved in mechanical stretch/leptin-induced VSMC hypertrophy, assessed by changes in wet weight and protein synthesis by measuring [^3^H]-leucine incorporation**. Mechanical stretch for 3 days significantly increased RPV wet weight **(A)** and protein synthesis **(B)**, while pre-treatment with the calcineurin inhibitor FK506 (FK; 0.1 and 1 nM) reduced the stretch-induced change in wet weight **(A)** and protein synthesis **(B)** (*n* = 6–8). ^*^p < 0.05 vs. unstretched. #*p* < 0.05 vs. St at 3 days. Leptin (3.1 nM) treatment alone significantly increased wet weight **(C)** and protein synthesis **(D)** while FK506 (0.1 and 1 nM) resulted in a reduction in hypertrophy as evaluated by wet weight changes **(C)** and protein synthesis **(D)** (*n* = 6–8). ^*^p < 0.05 vs. untreated. ^#^*p* < 0.05 vs. Lep at 3 days.

We have previously shown that mechanical stretch induces VSMC hypertrophy and that leptin is involved in this process (Zeidan et al., 2005; Ghantous et al., 2015b). To study whether calcineurin is implicated in mechanical stretch-induced VSMC hypertrophy, RPVs were pre-treated with the calcineurin inhibitor FK506 (0.1 or 1 nM) and mechanically stretched for 3 days. Hypertrophy was assessed by wet weight changes and protein synthesis (measured by [^3^H]-leucine incorporation). Mechanical stretch alone, without FK506 addition, significantly increased both wet weight and protein synthesis (Figures 5A,B). On the other hand, FK506 (1 nM) alone, without stretch, had no effect on wet weight and leucine incorporation (Figures 5A,B). However, RPVs treated with 0.1 nM FK506 and stretched for 3 days showed reduced wet weight change and protein synthesis, compared to stretched RPVs, whereas 1 nM FK506 resulted in a significant attenuation of hypertrophy (Figures 5A,B). It's important to note that the relationship between wet and dry weights of RPVs in the different groups showed no significant differences in the wet weight/dry weight ratios between the different groups, demonstrating that the increase in RPV wet weight was not due to increased water retention (data not shown). These data suggest that calcineurin plays a pivotal role in mechanical stretch-induced VSMC hypertrophy.

Our previous studies revealed that leptin is implicated in mechanical stretch-induced VSMC hypertrophy (Zeidan et al., 2005; Ghantous et al., 2015b). To study whether calcineurin is involved in leptin-mediated VSMC hypertrophy, exogenous leptin (3.1 nM) was added to RPVs for 3 days with or without FK506 (0.1 or 1 nM), followed by analysis of wet weight changes and protein synthesis. Leptin alone significantly increased RPV wet weight and protein synthesis (Figures 5C,D). FK506 (1 nM) alone had no effect on RPV hypertrophy (Figures 5C,D). Treatment with both leptin and 0.1 nM FK506 reduced the change in wet weight and protein synthesis, while 1 nM FK506 significantly attenuated VSMC hypertrophy by decreasing the leptin-induced increase in protein synthesis and significantly reducing wet weight change (Figures 5C,D). Thus, calcineurin is involved in leptin-mediated VSMC hypertrophy.

### Involvement of calcineurin activation in mechanical stretch-mediated leptin synthesis and release

**Figure 6:**
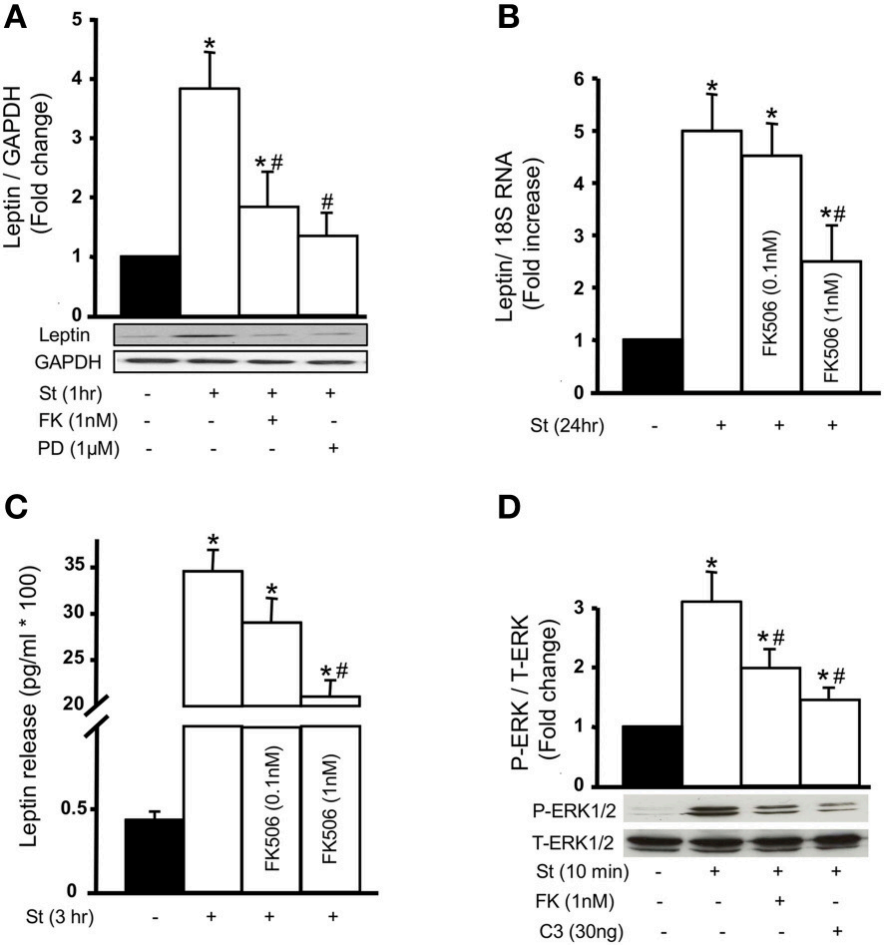
**Calcineurin activation promotes mechanical stretch-induced leptin production and release as well as ERK1/2 phosphorylation. (A)** Leptin protein expression was evaluated using Western blot densitometric scans and normalized to unstretched RPVs. Pre-treatment with 1 nM FK506 (FK) followed by 1 h of mechanical stretch significantly decreased leptin protein expression. The ERK1/2 inhibitor PD98059 (1 μM) also significantly reduced leptin expression in response to 1 h of mechanical stretch (*n* = 5–6). ^*^p < 0.05 vs. unstretched. #*p* < 0.05 vs. St 1 h. **(B,C)** Leptin release into the culture medium by VSMCs was measured using immunometric assay while leptin mRNA expression was measured using Real-Time PCR analysis. Mechanical stretch for 24 h significantly increased leptin secretion and mRNA expression, while FK506 (FK; 0.1 and 1 nM) reduced stretch-mediated leptin release **(B)** and mRNA expression **(C)** (*n* = 8–9). ^*^p < 0.05 vs. unstretched. #*p* < 0.05 vs. St 24 h. **(D)** ERK 1/2 phosphorylation (P-ERK1/2) was evaluated by Western blot and normalized to Total-ERK (T-ERK1/2) and to the unstretched RPVs. Mechanical stretch for 10 min significantly increased P-ERK1/2 compared to unstretched RPVs. FK506 (1 nM) and C3 (30 ng/mL) each significantly reduced ERK1/2 phosphorylation in response to 10 min of mechanical stretch (*n* = 5). ^*^p < 0.05 vs. unstretched. ^#^*p* < 0.05 vs. St 10 min.

To study the role of calcineurin on the expression of leptin protein, 1 nM FK506 was used followed by mechanical stretch for 1 h. Calcineurin inhibition by FK506 significantly reduced mechanical stretch-induced leptin protein expression (Figure 6A), further validating and confirming calcineurin's role in leptin synthesis in response to stretch.

To investigate whether the increased expression of leptin in response to mechanical stretch was caused by the direct production of leptin, and whether calcineurin is involved in this process, Real-Time PCR analysis was performed to study leptin mRNA expression after 24 h of stretch. Mechanical stretch alone significantly upregulated leptin mRNA expression, while 0.1 nM FK506 reduced stretch-induced leptin mRNA expression (Figure 6B). Pre-treatment with 1 nM FK506 significantly decreased leptin mRNA expression in response to mechanical stretch, indicating that calcineurin is involved in mechanical stretch-induced leptin expression at the transcriptional level.

Mechanical stretch induces the release of leptin into culture media by VSMCs after 1–3 days of stretch (Zeidan et al., 2005). To study whether calcineurin activation affects leptin release in response to mechanical stretch, RPVs were stretched for 3 h with or without FK506 (0.1 or 1 nM). Leptin was then measured in the culture media using immunometric assay. Mechanical stretch alone significantly increased leptin release into the media (Figure 6C) whereas pre-treatment with 1 nM FK506 significantly decreased mechanical stretch-induced leptin release, to an even greater extent than 1 nM FK506 (Figure 6C).

We have previously shown that the MAP kinase ERK1/2 is involved in mechanical stretch-induced VSMC hypertrophy (Zeidan et al., 2000, 2003a). However, it is still unclear whether ERK 1/2 affects leptin protein expression in response to mechanical stretch. To study the involvement of ERK1/2 in this process, RPVs were pre-treated with the selective ERK inhibitor PD98059 (1 μM) and stretched for 1 h, followed by Western blot analysis for leptin protein expression. Mechanical stretch-induced leptin protein expression was significantly decreased by PD98059 (Figure 6A), indicating that ERK1/2 is involved in leptin protein expression.

### Involvement of the RhoA/ROCK pathway and calcineurin on mechanical stretch-induced ERK 1/2 activation

Both calcineurin activation (Figure 3) and ERK1/2 phosphorylation are involved in mechanical stretch-induced VSMC hypertrophy (Zeidan et al., 2000, 2003a). However, whether there is a cross talk between these pathways in response to mechanical stretch is unclear. To investigate this, RPVs were pre-treated with 1 nM FK506 followed by stretch for 10 min and ERK1/2 phosphorylation was evaluated by Western blot. Stretch alone significantly upregulated ERK1/2 phosphorylation (Figure 6D) whereas calcineurin inhibition by FK506 significantly reduced stretch-induced p-ERK1/2 (Figure 6D), indicating that calcineurin activation is upstream to ERK phosphorylation.

To study whether the RhoA/ROCK pathway affects ERK 1/2 phosphorylation, RPVs were treated with C3 (30 ng/mL) and stretched for 10 min. Inhibition of RhoA by C3 resulted in a significant reduction in p-ERK1/2 by mechanical stretch (Figure 6D), suggesting that the RhoA/ROCK pathway is involved in mechanical stretch-mediated ERK1/2 activation.

## Discussion

**Figure 7:**
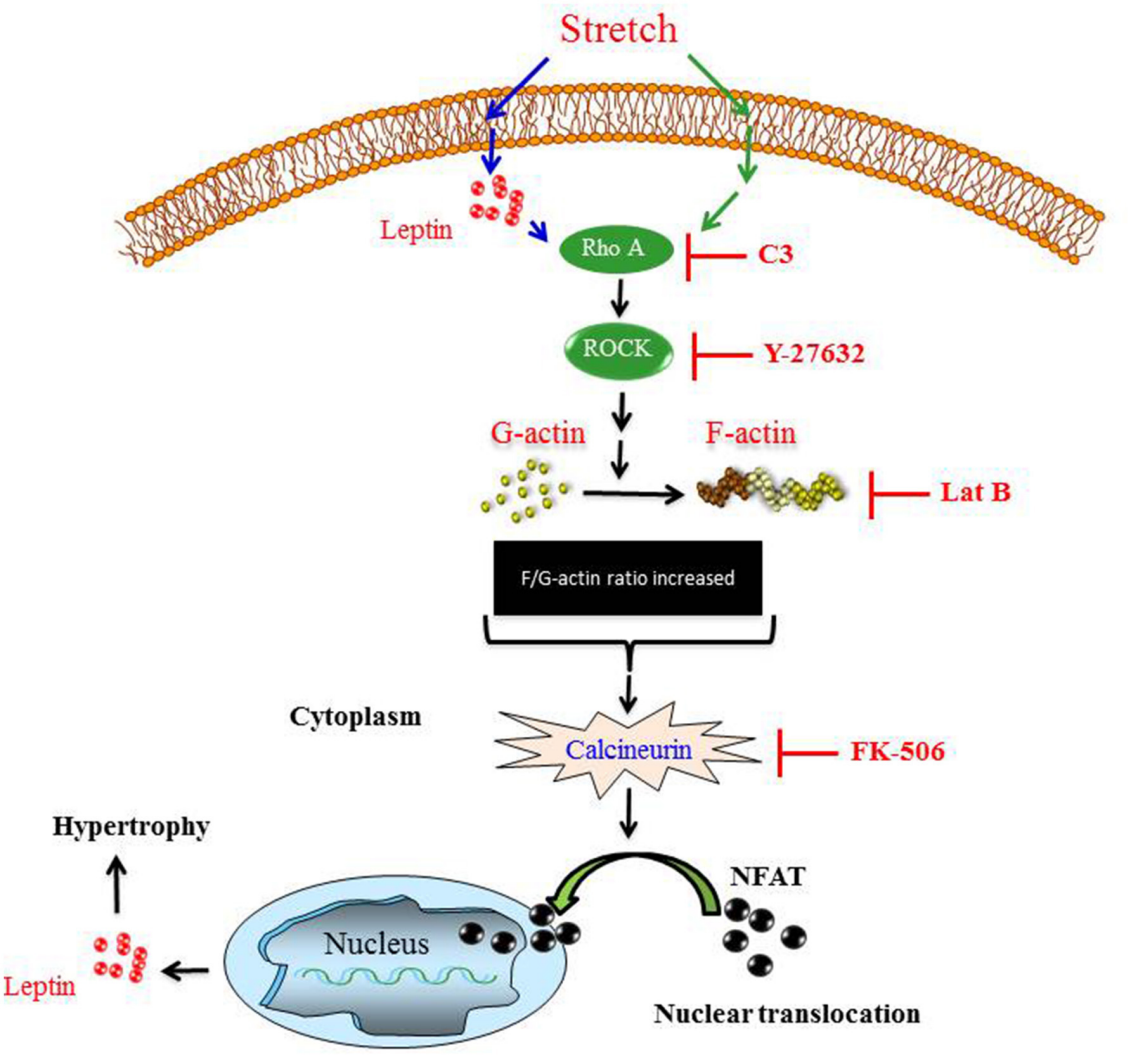
**The proposed mechanism of mechanical stretch-induced VSMC hypertrophy demonstrates that the RhoA/ROCK pathway activation, calcineurin activation, NFAT nuclear translocation, and leptin synthesis are involved in this process**.

In the present study, we investigated the involvement of Ca^2+^ and the calcineurin/NFAT signaling pathway in mechanical stretch-induced VSMC hypertrophy and leptin synthesis and secretion in VSMCs. The major and novel findings of this study are: (1) Ca^2+^ inflow is involved in mechanical stretch-mediated leptin synthesis from VSMCs; (2) Mechanical stretch/Leptin-induced activation of calcineurin promotes the nuclear translocation of NFAT and expression of MCIP1; (3) The RhoA/ROCK pathway and an intact actin cytoskeleton are required for mechanical stretch/leptin-induced calcineurin activation; (4) Calcineurin plays a pivotal role in mechanical stretch/leptin-induced VSMC hypertrophy. These findings suggest that the calcineurin/NFAT pathway is involved in the mechanisms that lead to leptin synthesis in VSMCs in response to mechanical stretch (Summarized in Figure 7).

Hypertension has been implicated in promoting vascular hypertrophy (Malmqvist and Arner, 1988, 1990; Zeidan et al., 2000, 2003a,b, 2005; Intengan and Schiffrin, 2001; Shyu, 2009; Ren et al., 2010; Turczynska et al., 2012). Extensive research has been done to decipher the mechanisms by which hypertension induces VSMC hypertrophy. The major extracellular mediator of hypertension-induced VSMC hypertrophy is thought to be mechanical stretch, which activates several signaling pathways. Among these pathways are the RhoA/ROCK pathway (Ghantous et al., 2015b), MAPK activation (Zeidan et al., 2003a), and ROS formation (Ghantous et al., 2015b). However, evidence also suggests that hypertension mediates cardiac hypertrophy through calcineurin/NFAT signaling pathways (Bueno et al., 2002a; Takeda et al., 2002).

Recently, we have reported that mechanical stretch significantly increases intracellular leptin levels and enhanced leptin release in VSMCs (Ghantous et al., 2015b). In the mechanism of mechanical stretch-mediated increase in leptin protein expression, we hypothesized that leptin is directly synthesized by VSMCs in response to mechanical stretch and not pre-stored inside the cells and released upon stimulation. To test this hypothesis, leptin mRNA synthesis (transcription) and protein synthesis (translation) were inhibited by actinomycin D and cycloheximide, respectively. Both inhibitors significantly inhibited leptin protein synthesis and secretion. Our results demonstrated that mechanical stretch induces mRNA and protein synthesis of leptin in VSMCs.

Many studies have shown a clear relationship between Ca^2+^ homeostasis and various VSMC functions and signaling, including VSMC growth and contraction (Waitkus-Edwards et al., 2002; Ren et al., 2010). Indeed, it is well-established that Ca^2+^-dependent signaling molecules underlie the hypertrophic program and contribute to the pathogenesis of vascular remodeling. A study by Ren et al. has shown that the hypertrophic effect of Ca^2+^ influx was through L-type calcium channels in a stretched RPV. In their study, they showed that Ca^2+^ influx induced the expression of contractile phenotype marker genes via the RhoA/ROCK cascade (Ren et al., 2010). Our data demonstrate the involvement of L-type calcium channels in mechanical stretch-induced VSMC hypertrophy and leptin synthesis and release, as suggested by the effect of L-type calcium channel blocker nifedipine. Indeed, our results demonstrated that nifedipine at a low concentration of 1 μM did not affect mechanical stretch-induced VSMC hypertrophy, whereas a concentration of 10 μM significantly inhibited this effect. This indicates an involvement of L-type calcium channels in the extracellular Ca^2+^ influx and VSMC hypertrophy induced by mechanical stretch. To date and to our knowledge, there are no studies that have examined the role of L-type calcium channels in mechanical stretch-induced leptin synthesis and secretion from VSMCs. Interestingly, we found that 10 μM nifedipine significantly inhibited mechanical stretch-induced leptin synthesis and secretion from the RPV. This suggests that an elevated intracellular Ca^2+^ concentration provides a major contribution to leptin synthesis evoked by mechanical stretch.

To evaluate the downstream signaling of Ca^2+^ in relation with hypertrophy and leptin synthesis in VSMCs, we investigated the involvement of the calcineurin/NFAT pathway in this process. Increased intracellular Ca^2+^ in the cytosol leads to calcineurin activation (Crabtree, 2001; Hogan et al., 2003). Calcineurin is a cytoplasmic Ca^2+^-regulated phosphatase which has been shown to play a critical role in vascular and cardiac remodeling (Bueno et al., 2002b). Various hypertrophic factors including leptin (Rajapurohitam et al., 2012), pressure, and alpha-adrenergic agonist have been shown to activate calcineurin in cardiomyocytes [reviewed by Wilkins and Molkentin (2004)]. Indeed, increased activity of calcineurin is associated with massive heart hypertrophy that often results in congestive heart failure (Molkentin et al., 1998). Rauch and Loughna showed that stretch-induced ERK phosphorylation in the C_2_C_12_ cell line is mediated by calcineurin activation (Rauch and Loughna, 2008).

To our knowledge, there have been no studies investigating the involvement of calcineurin on mechanical stretch-induced VSMC hypotrophy and leptin synthesis. In the present study, we observed that 10 μM nifedipine significantly inhibited both mechanical stretch- and exogenous leptin- induced calcineurin activation, indicating that calcineurin activation is mediated by Ca^2+^ entry. Our results, obtained using both calcineurin phosphatase activity and mRNA expression of MCIP1, an indicator of calcineurin activity, show that mechanical stretch/leptin-induced VSMC hypertrophy is associated with calcineurin activation. Rajapurohitam et al. have shown the ability of leptin to induce calcineurin activation using cultured neonatal rat ventricular myocytes (Rajapurohitam et al., 2012). It is firmly established that the specific inhibitor for calcineurin FK506 is a potent inhibitor of calcineurin activation (Martínez-Martinez and Redondo, 2004; Roehrl et al., 2004). In this study, we used FK506 to determine the need of calcineurin activity in mechanical stretch-induced leptin synthesis and VSMC hypertrophy. In agreement with this concept, we found that pre-treatment with FK506 significantly inhibited both mechanical stretch- and exogenous leptin-induced RPV hypertrophy. These results suggest that calcineurin activation is a crucial step in the action of mechanical stretch and exogenous leptin.

To further validate the above findings, we investigated the downstream effectors of calcineurin signaling, including NFAT transcription factor. In this study, we found that calcineurin activity was associated with nuclear translocation of NFAT in response to 3.1 nM leptin. Calcineurin was found to peak at 30 min following initiation of mechanical stretch or addition of leptin, followed by an increase in NFAT nuclear translocation as assessed by fluorescence microscopy at 60 min after leptin addition. Indeed, leptin addition for 60 min promoted almost complete translocation of NFAT into the nucleus, indicating that leptin-induced activation of calcineurin promotes the activation of NFAT. It is important to mention that mechanical stretch activated calcineurin more than leptin did. Since mechanical stretch activates a wide range of signaling pathways (Lehoux and Tedgui, 1998; Zeidan et al., 2005; Seo et al., 2013), there may be other mediators, besides leptin, that are activating calcineurin. It has been previously demonstrated that calcineurin directly dephosphorylates cytoplasmic NFAT and permits translocation of NFAT into the nucleus (Rao et al., 1997; Kudryavtseva et al., 2013). NFAT regulated genes have subsequently been shown to modulate different functions of VSMCs such as myocyte enhancer factor 2 (MEF2) and GATA-4 (Wilkins and Molkentin, 2004). Recent studies have also revealed roles for NFAT activation in VSMC hypertrophy and proliferation induced by pulmonary hypertension (Bierer et al., 2011; Hou et al., 2013). Moreover, transgenic mice that overexpress active calcineurin or NFATc4 in the heart develop massive cardiac hypertrophy (Molkentin et al., 1998). Furthermore, deletion of NFATc2 gene has been shown to significantly inhibit pressure overload-induced heart hypertrophy (Bourajjaj et al., 2008).

NFAT binds to GATA-4 and activates the transcription of several hypertrophic genes (Molkentin et al., 1998; Liang and Gardner, 1999; Saadane et al., 1999; Babu et al., 2000; Hsieh et al., 2015). The role of GATA-4 in promoting hypertrophy has been well-established in the cardiovascular system (Chien et al., 1991; Saadane et al., 1999; Hsieh et al., 2015). Indeed, several studies have shown the ability of GATA-4 to activate several hypertrophic gene expressions like b-MHC and c-fos (Herzig et al., 1997; Oka et al., 2006). We have previously demonstrated that GATA-4 activation represents a critical role for mediating mechanical stretch-induced VSMC hypertrophy (Ghantous et al., 2015b). These data suggest that NFAT is involved in mechanical stretch-induced GATA-4 activation.

To further explore the possible mechanisms of mechanical stretch/leptin-induced calcineurin activation, we investigated the involvement of the RhoA/ROCK pathway and intact actin cytoskeleton dynamics. The importance of the Rho family of small GTPases and the actin cytoskeleton dynamics in controlling VSMC remodeling has long been appreciated (Jalil et al., 2005; Nelson et al., 2005; Zeidan et al., 2006, 2007). Indeed, several studies have shown that hypertrophic growth of cardiac and vascular cells is associated with cellular morphological changes and increased F-actin/G-actin ratio (Nelson et al., 2005; Zeidan et al., 2006, 2007). We have recently shown that the RhoA/ROCK pathway is an important mediator for mechanical stretch-induced actin cytoskeletal dynamics leading to VSMC hypertrophy (Ghantous et al., 2015b).

An active RhoA/ROCK pathway is characterized by an increased F-actin/G-actin ratio. To study the effect of inhibiting the RhoA/ROCK pathway on calcineurin activation in response to mechanical stretch or leptin, we used the selective Rho inhibitor clostridial toxin C3 exoenzyme, the selective ROCK inhibitor Y-27632, and the actin depolymerization agent latrunculin B. Our data demonstrate that all of these compounds significantly attenuated mechanical stretch/leptin-induced calcineurin activation, indicating that mechanical stretch/leptin-induced activation of calcineurin is mediated by the RhoA/ROCK pathway and requires an intact actin cytoskeleton. Moreover, inhibiting the RhoA/ROCK pathway attenuated leptin-induced NFAT translocation in RASMCs. Thus, in accordance with published data, the current study demonstrated that RhoA pathway and low G-actin/F-actin ratio mediated VSMC hypertrophy is dependent on the calcineurin/NFAT signaling pathway.

To further validate the above findings, the involvement of ERK1/2 activation in mechanical stretch-induced VSMC hypertrophy was investigated. ERK1/2 pathway has been implicated as a positive regulator for vascular hypertrophy (Zeidan et al., 2000). In fact, mechanical stretch and exogenous leptin have been shown to stimulate vascular hypertrophy, generally via ERK1/2 activation (Zeidan et al., 2000, 2005). In this study, our results demonstrated that mechanical stretch-induced ERK1/2 activation was attenuated by both FK506 and C3 exoenzyme. Therefore, these data indicate that calcineurin and the RhoA/ROCK pathway mediate mechanical stretch-induced ERK1/2 activation.

There are some limitations in this study. First, this form of *ex vivo* organ culture uses an isolated blood vessel instead of an *in vivo* experimental procedure. However, we used this model to study the effect of hypertension and leptin in an isolated setting without the effects of the central nervous system and the effects of leptin on the sympathetic nervous system. Second, the data acquired from a venous vessel may not necessarily reflect those in other vascular tissues. However, the results could be of direct application to portal hypertension, and this model of mechanically stretching the RPV has been well-characterized in many publications (Zeidan et al., 2000, 2003a,b, 2005; Ren et al., 2010; Turczynska et al., 2012). The RPV has also been used in experiments of partial occlusion in order to study the effects of increased RPV pressure (Malmqvist and Arner, 1988, 1990).

In summary, our results, for the first time, demonstrate that the calcineurin/NFAT pathway is involved in mechanical stretch-induced VSMC remodeling and leptin synthesis. Moreover, our study showed that the RhoA/ROCK pathway and intact actin cytoskeleton are required for mechanical stretch-induced calcineurin activation.

## Author contributions

NS, CMG, ZF and AZ contributed in generating experimental data. NS, CMG, WNS, KZ, and AZ contributed in discussion and reviewed/edited manuscript. CMG, NS, and AZ wrote the manuscript and drew the figures.

### Conflict of interest statement

The authors declare that the research was conducted in the absence of any commercial or financial relationships that could be construed as a potential conflict of interest.
